# Neuropsychiatric Symptom Phenotype Prevalence in Diverse Older Adults with Alzheimer's Disease

**DOI:** 10.1002/alz70857_107007

**Published:** 2025-12-25

**Authors:** Alyssa N. De Vito, Colin Stein, Masood Manoochehri, Pamela Del Rosario, Jiji T. Kurup, Ajneesh Kumar, Nicholas R. Ray, Timothy J. Hohman, Michael L Cuccaro, Gary W Beecham, Christiane Reitz, Edward D. Huey, ADSP NPS Harmonization Working Group

**Affiliations:** ^1^ Brown University Medical School, Providence, RI, USA; ^2^ Department of Psychiatry and Human Behavior, Alpert Medical School of Brown University, Providence, RI, USA; ^3^ Columbia University Irving Medical Center, New York, NY, USA; ^4^ Vanderbilt Memory and Alzheimer's Center, Vanderbilt University School of Medicine, Nashville, TN, USA; ^5^ Dr. John T. Macdonald Foundation Department of Human Genetics, University of Miami Miller School of Medicine, Miami, FL, USA; ^6^ Department of Biostatistics and Data Science, Wake Forest School University of Medicine, Winston‐Salem, NC, USA

## Abstract

**Background:**

Published rates of neuropsychiatric symptoms (NPS) vary widely (30‐85%); This inconsistency may be driven by heterogeneity in demographics, clinical characteristics, and NPS evaluation methods. A robust understanding of clinico‐demographic factors that influence NPS prevalence is needed. This project is part of a larger effort (U01AG07950) aimed at harmonizing NPS phenotype data from the Alzheimer's Disease Sequencing Project (ADSP) to provide more accurate data on the prevalence of NPS.

**Method:**

Using data from eight ADSP studies (*n* = 36,194), we generated three NPS phenotypes: early (EPA)‐ and late psychosis/agitation (LPA), and affective symptoms (AS). EPA was defined by a non‐zero score on Delusion, Hallucination, or Agitation domains on the Neuropsychiatric Inventory Questionnaire (NPI‐Q) when the participant's global Clinical Dementia Rating (CDR®) score was 0.5 or 1. LPA used these same symptom thresholds but when CDR® score ≥ 2. Psychosis/agitation (PA+) phenotype was defined as having EPA or LPA at any study visit. The affective symptoms phenotype (AS+) required a non‐zero score on the NPI‐Q Depression, Anxiety, or Irritability domains, or meeting the threshold for depression on the Center for Epidemiological Studies‐Depression (CES‐D) or Geriatric Depression Scale (GDS), regardless of CDR score. Descriptive statistics and chi‐square analyses were utilized to explore prevalence of symptoms and differences by demographics.

**Result:**

18.8% met criteria for the AS phenotype and 5.2% individuals met criteria for the PA phenotype. 82.9% of PA+ individuals were classified as EPA phenotype and 17.1% being classified as the LPA phenotype. Prevalence of AS+ and PA+ phenotypes differed by race (*p's* < .001). AS+ was most prevalent in individuals who identified as American Indian/Alaskan Native (79.4%) or more than one race (78.6%). PA+ was most prevalent in individuals who self‐identified as more than one race (43.0%) or Asian/Pacific Islander (23.4%).

**Conclusion:**

Prevalence of the AS phenotype was higher than the PA phenotype in the overall sample. Among PA+ individuals, symptoms were more likely to occur early in the disease. Prevalence of AS and PA phenotypes differed by race, suggesting the need for further research in individuals of diverse ancestries to examine ancestry‐specific genetic determinants, molecular pathways, and drug targets to develop tailored treatments.

